# Recent innovations in membrane-protein structural biology

**DOI:** 10.12688/f1000research.16234.1

**Published:** 2019-02-22

**Authors:** James P Allen

**Affiliations:** 1 School of Molecular Sciences, Arizona State University, Tempe, AZ, USA

**Keywords:** protein crystallography, cryo-electron microscopy, X-ray free electron laser, protein dynamics

## Abstract

Innovations are expanding the capabilities of experimental investigations of the structural properties of membrane proteins. Traditionally, three-dimensional structures have been determined by measuring x-ray diffraction using protein crystals with a size of least 100 μm. For membrane proteins, achieving crystals suitable for these measurements has been a significant challenge. The availabilities of micro-focus x-ray beams and the new instrumentation of x-ray free-electron lasers have opened up the possibility of using submicrometer-sized crystals. In addition, advances in cryo-electron microscopy have expanded the use of this technique for studies of protein crystals as well as studies of individual proteins as single particles. Together, these approaches provide unprecedented opportunities for the exploration of structural properties of membrane proteins, including dynamical changes during protein function.

## Introduction

**Figure 1.**
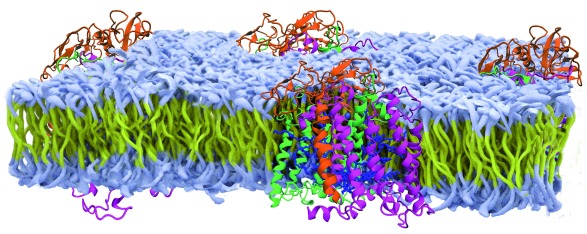
Model of the bacterial reaction center buried within a membrane. Shown are the protein subunits of the reaction center from *Rhodobacter sphaeroides* (orange, green, purple subunits with blue cofactors) that are positioned within an artificial membrane (yellow carbon chains with white head groups) based upon a range of spectroscopic studies (modified from 1).

Membrane proteins are essential components of cell membranes, playing key roles in a wide range of cellular processes. Membrane proteins are often composed of multiple protein subunits, with some regions being hydrophobic and buried within the cell membrane whereas others are hydrophilic and exposed to the solvent ^1^ ( Figure 1). Spanning the cell membrane is essential to their function, for example G protein–coupled receptors (GPCRs) sense alterations in the environment and transmit responses across cell membranes as part of signal transduction pathways. In photosynthesis, reaction centers convert light energy into chemical energy through electron and proton transfer events, creating proton gradients across the membrane. The proton gradients, which are produced in photosynthesis and processes such as respiration, are used by ATP synthase to synthesize the energy-rich molecule ATP. Owing to their intrinsic roles in metabolism, membrane proteins with mutations and improper folding are associated with impaired metabolic processes, which often result in diseases. Thus, in addition to facilitating our understanding of the fundamental science, new structures of membrane proteins provide a platform for rational drug design ^2, 3^.

**Figure 2.**
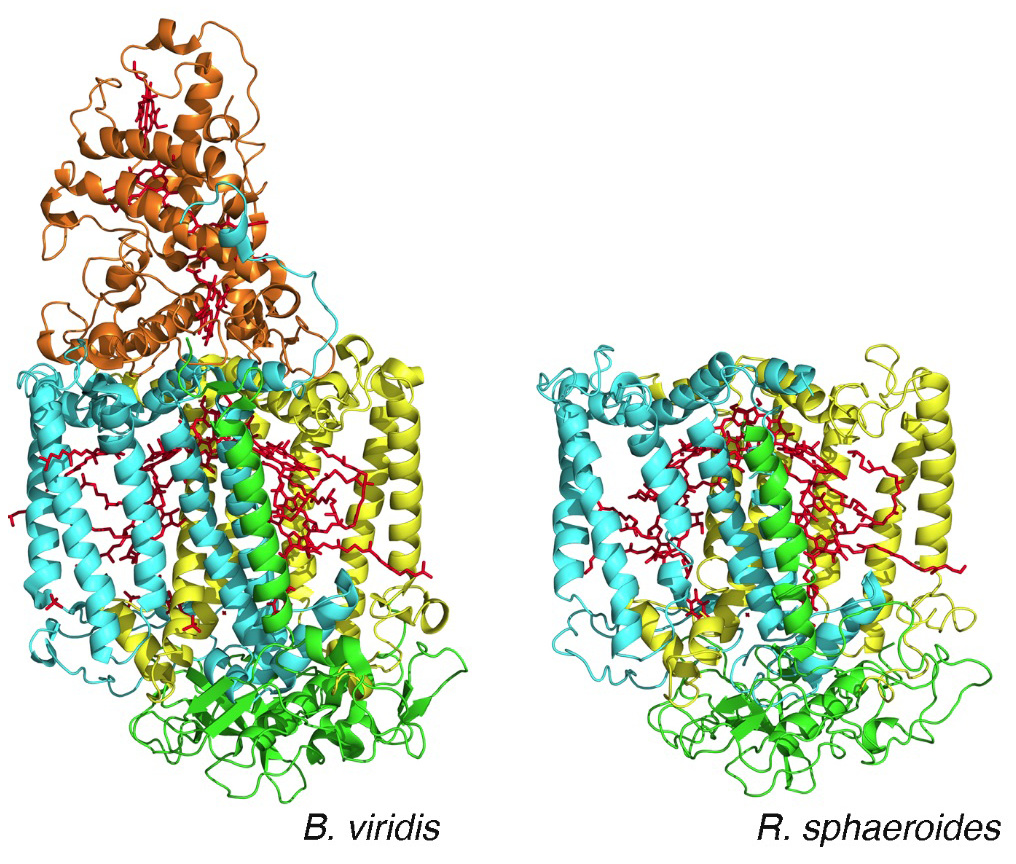
Three-dimensional structures of reaction centers from *Blastochloris viridis* and *Rhodobacter sphaeroides* The arrangement of the bacteriochlorophyll and quinone cofactors (red) is shown. The two core protein subunits—L (yellow) and M (cyan)—and the H subunit (green) are all conserved, whereas the tetraheme cytochrome subunit (orange) is found only in reaction centers from *B. viridis*. (PDB files 1PRC and 4RCR ^5, 6^.)

Since the 1950s, the technique of protein crystallography has been the primary choice for the determination of the structures of proteins. Initially, the resulting structures had been restricted to water-soluble proteins, excluding proteins that are embedded in cell membranes. Extraction of proteins from the cell membrane traditionally made use of detergents and the resulting protein–detergent complexes were regarded as being biochemically too difficult to crystallize. Electron microscopy provided an opportunity to perform structural studies on membrane proteins that formed two-dimensional crystals in membranes, allowing measurement of both image and diffraction data. These studies provided the initial structural information for membrane proteins, highlighted by the determination of the overall structure of bacteriorhodopsin and the presence of seven-transmembrane helices in 1975 ^4^. In 1985, the landmark report of the structure of the reaction center from *Rhodopseudomonas viridis*, later renamed *Blastochloris viridis*, demonstrated the feasibility of obtaining diffraction-quality crystals of membrane proteins, and the authors Deisenhofer, Michel, and Huber received the Nobel Prize in 1988 ^5^. This work was followed by additional structures of other membrane proteins, including the reaction center from *Rhodobacter sphaeroides* in 1987 ^6^. The reaction center structures established many aspects of membrane proteins, such as the prediction that the long regions of hydrophobic amino acid residues evident in their sequences formed long membrane-spanning helices ( Figure 2). In addition to being very robust to genetic modifications, reaction centers after purification can be re-incorporated into membranes composed of a range of lipids and non-lipid polymer membranes ( Figure 1). Of particular note, the structures showed an unexpected symmetrical arrangement of the bacteriochlorophylls and quinone cofactors in two branches ^7^. The combination of structural and spectroscopic analysis established the role of the cofactor branches as electron transfer pathways and enabled the development of detailed electron transfer mechanisms that validated the theoretical ideas of Marcus ^8^, who won the Nobel Prize in 1992.

These efforts demonstrated the scientific benefit that could be gained from structural analysis of membrane proteins. Although the rewards were notable, significant technical obstacles remained, such as limited expression of the proteins and poor diffraction quality of the crystals. The tenacity of many research groups produced additional structures, but the number remained small compared with the tens of thousands of membrane proteins that are present in cells ^9^. This report reviews the exciting novel technical improvements in structural biology which are enhancing experimental research efforts to determine structures of membrane proteins and dynamical changes during catalysis.

## Structural biology of membrane proteins

Using x-ray diffraction requires the organization of the membrane proteins into crystalline arrays ^10^. The biochemical formation of crystals of membrane proteins remains a challenge, but new approaches are overcoming difficulties associated with crystallization. In this review, strategies and protocols for crystallization, including how membrane proteins can be modified to enhance their crystallization, are briefly described. Then an overview is provided concerning how small micron-sized crystals now can be used because of advances of micro-focus beams at synchrotrons and the introduction of x-ray free-electron lasers (XFELs) as an extremely bright source of x-rays. The enhanced capability of cryo-electron microscopy (cryo-EM) for structural measurements of protein crystals is presented along with consideration of the impact of such measurements on proteins as single particles.

### Protein crystallization

Protein crystallization developed along with x-ray diffraction as a means to determine three-dimensional structures, and many reviews that describe crystallization protocols are available ^11, 12^. Membrane proteins are sparingly soluble in aqueous solutions and require the use of detergents to produce a soluble protein–detergent–lipid complex. Since crystallization remains, by and large, an empirical procedure, requiring testing of thousands of possible biochemical conditions, the additional parameters associated with the detergents and inclusion of amphiphilic molecules significantly amplify the number of conditions that are typically tested. Highly automated instrumentation can manipulate protein solutions on the nanoliter scale to test large numbers of conditions.

An effective alternative to the approach of crystallizing protein–detergent complexes is the use of lipids in place of detergents ^13, 14^. Via the lipid cubic phase or *in meso* method, the lipids phase-separate from the solution, forming a bicontinuous cubic phase, which contains bilayers into which the isolated proteins can be reconstituted. The addition of a precipitant triggers alteration of the mesophase properties, which can lead to enrichment of the protein and crystallization. Crystallization screens are available using this approach, which has proven useful in the structure determination of hundreds of membrane proteins.

In addition to detergents and lipids, a wide range of solubilizing agents have been developed to extract proteins from the membranes while maintaining protein–lipid interactions. Examples include styrene maleic acid co-polymers that efficiently liberate membrane proteins, including large unstable membrane proteins, into nanometer-sized bilayer discs that are suitable for structural analysis using cryo-EM and x-ray diffraction ^15– 17^.

**Figure 3.**
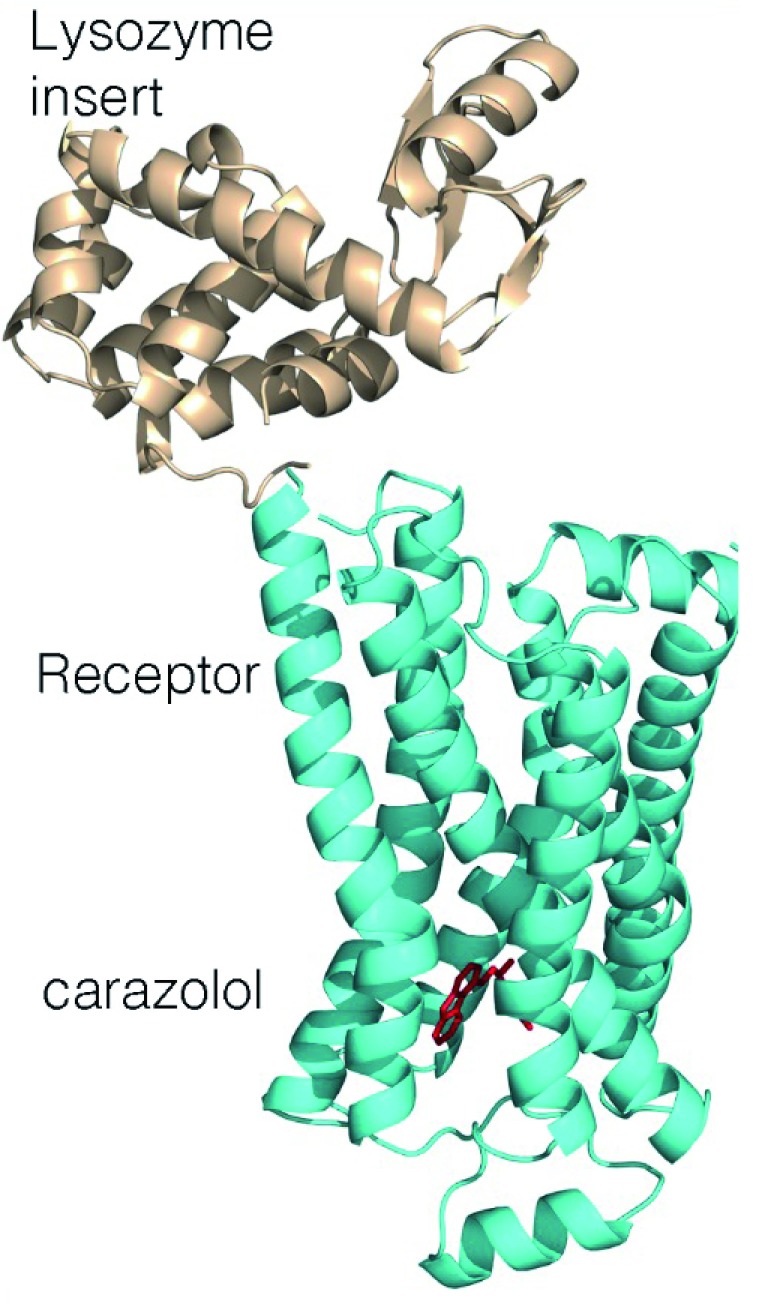
Three-dimensional structure of the human β _2_ adrenergic receptor (cyan) showing the presence of seven-transmembrane helices that are conserved among G protein–coupled receptors surrounding the agonist carazolol (red). For crystallization, a lysozyme domain (wheat) was added, replacing one of the connecting loops (PDB file 2RH1 ^21^).

Despite the fundamental role of GPCRs, progress in the elucidation of their structures lagged. Persistence in establishing suitable crystallization conditions for rhodopsin, the site for primary conversion of light in the signaling pathway that leads to vision, demonstrated both the feasibility and difficulties of crystallizing these receptors ^18, 19^. For the human β _2_ adrenergic receptor, diffraction-quality crystals were obtained only after modifications of the protein ^20^. Structures were independently obtained after the biochemical inclusion of a monoclonal antibody that binds at a surface loop and a genetic incorporation of a small water-soluble protein, T4 lysozyme, to produce a new domain favoring protein–protein interactions ( Figure 3) ^21, 22^. One of the authors of these papers, Kobilka, received the Nobel Prize in 2012 with Lefkowitz for their studies of GPCRs.

Improvements in crystallization through the use of thermostabilizing mutations, nanobodies, and novel fusion partners allow the crystallization of virtually any GPCR ^23^. These improvements have been accompanied by the development of x-ray beam lines at synchrotron sources ^24^. These sources are significantly more brilliant and more stable than previously available, providing a means to measure high-quality diffraction from crystals with sizes of tens of microns. The greater availability of these beams coupled with enhanced features, such as remote control of the instrumentation and remote data collection, is enabling new opportunities for structural studies.

### X-ray free-electron lasers

In 2000, Neutze *et al*. wrote a provocative theoretical paper describing a new technique for the measurement of diffraction from individual proteins ^25^. Rather than using x-ray beams from synchrotrons, they proposed to use x-ray beams produced using free-electron lasers that are more than 10 orders of magnitude brighter than the beams at synchrotrons. Such bright beams would quickly result in the destruction of the proteins, but they calculated that there would be a very short period of time, a few femtoseconds, when diffraction could be measured from nano-sized protein crystals. Since the crystals are destroyed immediately after measurement, only one still diffraction pattern could be recorded from each protein crystal. Obtaining the complete diffraction pattern would require measurement of diffraction from thousands of individual crystals that are each randomly oriented in the beam, which then are combined to generate the full diffraction data set. In 2009 and 2011, XFEL lines became available at x-ray facilities in the US and Japan, respectively, and experimental work soon thereafter clearly demonstrated the observation of diffraction that could be analyzed to produce full diffraction data sets for membrane proteins such as photosystem II ^26– 28^. Research continues to make rapid advances concerning many technical issues, such as methods for delivering nano-crystals to the beam and computational analysis of the large data sets, and the planned openings of new XFEL facilities promise increasing use of this technique ^29^.

### Cryo-electron microscopy

A new era in structural biology studies of membrane complexes is being ushered in by technical developments in cryo-EM. Images obtained from adenovirus by Dubochet’s laboratory demonstrated the possibility of measuring frozen unstained samples and obtaining images with a high level of detail ^30^. The structural work in Henderson’s laboratory on crystalline arrays of bacteriorhodopsin embedded in membrane sheets demonstrated the utility of cryo-EM for membrane proteins ^4, 31^. For their work in developing this field, Dubochet, Henderson, and Frank, who developed processing methods to sharpen the images, received the Nobel Prize in 2017. Since then, a number of advances, including improved methods for sample preparation, increasingly sensitive direct electron detectors, and advancements in image processing software, have helped overcome several challenges in its use ^32^. In crystallization experiments of conditions to grow protein crystals, they often only reach a very thin size (<400 nm) that is too small for traditional diffraction measurements. However, high-quality diffraction now can be obtained by using micro-electron diffraction (MicroED) ^33^.

When suitable crystals are not available, a new opportunity is available for structural analysis of membrane proteins using cryo-EM as improvements in instrumentation and image processing algorithms now allow structures to be obtained by single-particle analysis of images but without the need of crystals ^34^. The transient receptor potential (TRP) ion channel superfamily plays critical physiological roles and consequently is a drug target ^35^. Attempts to crystallize even small domains were unsuccessful, but the elucidation of several structures has been achieved by single-particle cryo-EM, revealing the arrangement of the protein in the membrane and providing insight into their roles in sensing heat and activating pain pathways ^36– 39^.

**Figure 4.**
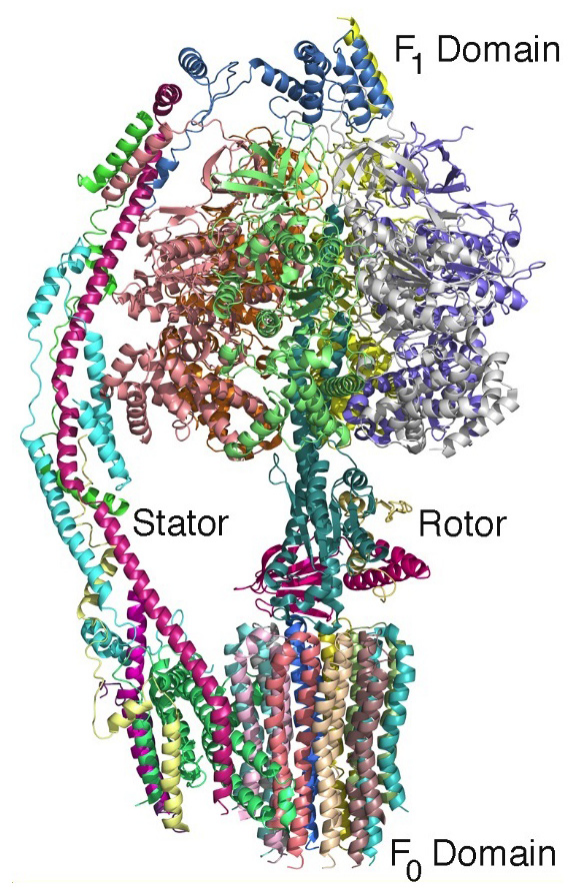
Three-dimensional structure of ATP synthase. The backbones of the 14 protein subunits are shown (and each protein subunit is colored differently). During catalysis, the hydrophilic F _1_ domain rotates relative to the domain that is embedded in the cell membrane. This rotation modifies the protein environment of the catalytic site, resulting in the production of ATP. The complex can be considered to be a rotary motor with a stator and rotor rotation that is powered by a transfer of protons across the cell membrane (PDB file 6CP6 ^41^).

The structure of ATP synthase serves as another excellent example of how cryo-EM can provide detailed structural information concerning large membrane-bound protein complexes. ATP synthase is essential to all of life as it produces the key molecule ATP using the energy stored in the form of a proton gradient across the cell membrane. This enzyme has two domains: a hydrophilic F _1_ domain that contains the catalytic sites and a membrane-embedded F _0_ domain. The structures of the intact complexes at atomic detail reveal how these domains are connected by additional protein components, termed the rotor and stalk, in analogy to mechanical rotary motion ^40, 41^ ( Figure 4). During catalysis, the transfer of protons through the F _0_ domain drives the rotation of the F _1_ domain relative to the F _0_ domain. This mechanical motion is coupled with structural changes at the catalytic sites that drive the synthesis of ATP. Together, these examples show the potential of cryo-EM to be a game changer for structural studies of large membrane proteins.

Cryo-EM has been used for the structure determination of biological complexes with molecular masses up to megadaltons, but lower molecular masses represent a technical challenge ^42^. The use of volta phase plates ^43^ has recently opened new possibilities in cryo-EM for the structure determination of membrane proteins that have molecular weights of only 110 to 120 kDa. This has revolutionized the field of GPCR research, where in the last two years nine structures of GPCRs coupled to heterotrimeric G proteins have been determined; by comparison, only one structure was determined by x-ray crystallography over the previous eight years ^44^.

## Membrane protein dynamics

Biochemical reactions typically occur on a millisecond timescale, requiring specific strategies for the experimental identification of any intermediates during the processes. Formation of intermediate configurations facilitates catalytic reactions, and identification of these rearrangements is needed to validate specific chemical mechanisms. Förster resonance energy transfer provides an experimental means to characterize dynamical changes of proteins in solution. In this technique, the proteins are typically labeled with dyes at specific locations, and the extent of energy transfer between the dyes is measured by using optical spectroscopy. Because the energy transfer has a pronounced dependence on the distance between the dyes, any conformational changes of the protein are revealed as alterations of the extent of energy transfer. While measurements of the average properties of proteins in solutions can be revealing, the ability to examine single molecules has paved the way to not only resolve features such as structural alterations upon ligand binding but also investigate interactions involving membrane proteins as occurs in fundamental cellular processes such as membrane transport and the rotation of ATP synthase during catalysis ^45^.

**Figure 5.**
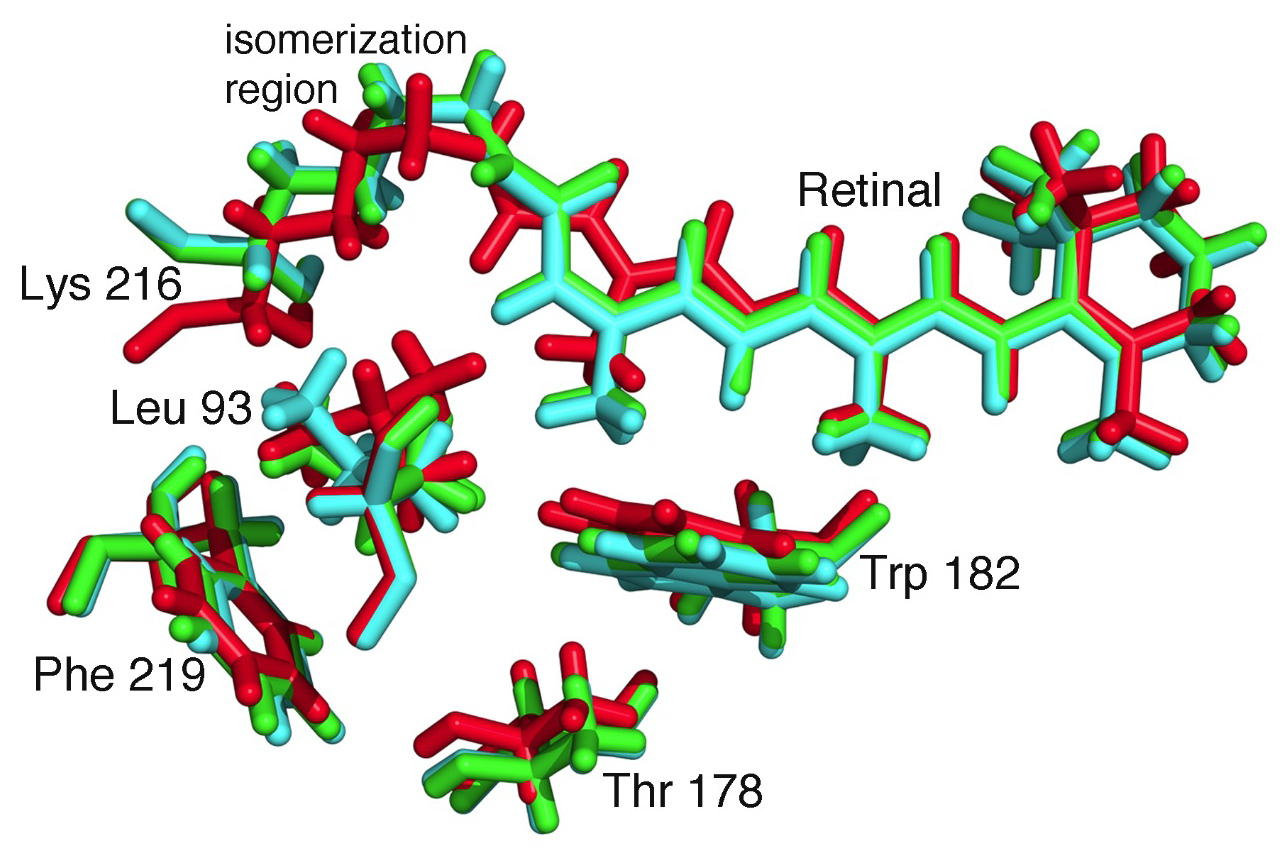
Three-dimensional structures of bacteriorhodopsin at different periods of time following illumination. Absorption of light by the retinal results in a trans-cis isomerization, which starts the process of proton transfer. Structural changes are evident on very fast timescales. The structures are shown for the retinal and a few surrounding residues, Leu 93, Thr 178, Trp 182, Lys 216, and Phe 219 at the initial time (red), 760 ns after excitation (green), and 1.7 ms after excitation (cyan). (PDB files 5B6V, 5B6X, and 5B6Z ^50^.)

The emerging capabilities for cryo-EM and x-ray diffraction are providing new strategies to map dynamical changes ^46^. Traditionally, protein crystallography has been used to probe such changes by trapping proteins in different functional states. The ability to measure very small crystals potentially opens the door to the examination of dynamical changes after the substrate has been introduced into suspensions containing nano-crystals of an enzyme ^47^. While this technique is undergoing experimental developments, time dependences of configurations involving intermediate catalytic configurations have been observed ^48, 49^. The power of time-resolved XFEL measurements has been demonstrated on proteins that can be optically triggered. As an example, the bacterium *Halobacterium halobium* grows in salty waters such as the Dead Sea with a distinctive purple color due to high concentrations of the membrane protein bacteriorhodopsin. This protein contains a retinal cofactor that undergoes a conformation change after light absorption, and the structural changes result in the transfer of a proton across the membrane in a few milliseconds. Whereas some conformational changes of this process have been identified by trapping experiments, the time dependence of the progression of conformations was measured using XFEL, showing how the motions are choreographed to achieve proton transfer ( Figure 5) ^50, 51^.

Another exciting direction for structural studies of membrane proteins is the dynamical interactions between proteins and lipids in cell membranes. Cell membranes have a multitude of different lipids that can flow within the membrane and form small microdomains containing enriched amounts of membrane proteins ^52^. Some membrane proteins depend upon interactions with specific lipids that can bind tightly to the protein, and interfacial lipids have both transient and stable interactions that stabilize membrane proteins, including GPCRs ^53, 54^.

## Concluding thoughts

Structural biology is evolving and new experimental avenues are becoming available for obtaining molecular information about membrane protein complexes. The techniques are continuing to be improved and new facilities are coming online, allowing structures to be determined from crystals that are increasingly small. These emerging innovations are promising for future research efforts and should have a major impact on our understanding of membrane proteins and their functions in cells.

## Abbreviations

Cryo-EM, cryo-electron microscopy; GPCR, G protein–coupled receptor; XFEL, x-ray free-electron laser.
